# Optimization of Sorghum Kafirin Extraction Conditions and Identification of Potential Bioactive Peptides

**DOI:** 10.1089/biores.2020.0013

**Published:** 2020-09-01

**Authors:** Tania P. Castro-Jácome, Luz E. Alcántara-Quintana, Erik G. Tovar-Pérez

**Affiliations:** ^1^ Instituto Tecnológico de Tepic. Av. Tecnológico No. 2595, Col. Lagos del Country, Tepic, Nayarit, México.; ^2^ Catedra CONACyT, Facultad de Enfermería y Nutrición, Universidad Autónoma de San Luis Potosí, Av. Niño Artillero No. 130, Zona Universitaria, S.L.P., México.; ^3^ Catedra CONACyT, Facultad de Ingeniería, Universidad Autónoma de Querétaro, Carretera Chichimequillas s/n, El Marqués, Querétaro, México.

**Keywords:** white sorghum, kafirins, amyloglucosidase, bioactive peptides

## Abstract

The interest in extracting kafirins (KAF), the main storage protein from sorghum grain has recently increased due to its gluten-free content and the significant scientific evidence showing the health benefits of the bioactive peptides from cereal grains in human diets. The objectives were to obtain the highest percentage of KAF extraction using amyloglucosidase as pretreatment to increase the extraction yield and predict the bioactive peptides in the KAF. In this study, pretreatments with amyloglucosidase increased the extraction yield of KAF compared with extraction methods using only ethanol and sodium metabisulfite. Two protein fragment sequences were identified from KAF extract and were evaluated for potential bioactive peptide using the BIOPEP-UWM database, which suggest that KAF proteins from white sorghum may be considered as good precursors of dipeptidyl peptidase-inhibitor, angiotensin-converting enzyme inhibitor, antioxidant and hypotensive peptides following chymotrypsin, thermolysin, and subtilisin and their combination. Average scores aligned using PeptideRanker confirmed KAF proteins' potential sources of bioactive peptides with over 5 peptides scored over 0.8. In addition, 31 unexplored peptide sequences that could have biological activity were identified. Our results suggest that KAF can be used in the peptide productions with potential biological activity and beyond.

## Introduction

Sorghum (*Sorghum bicolor* L. Moench) is the fifth most produced grain in the world Sorghum is a main grain in sub-Saharan Africa and South East Asia, with a global production of 61.4 million metric tons per year.^1,2^ This is a grain with many uses as raw material for livestock feed, food, brewing, and biofuels.^3^ Demand for sorghum is expected to increase in the midst of global climate changes, this grain is drought tolerant, viable for nonirrigated and semiarid farming areas, and could also be a potential alternative to wheat and maize.^2,3^ However, sorghum protein and starch has low digestibility, so its use in food is limited.^2,3^

Sorghum grain represents an average of 11% protein, which are classified into albumins, globulins, and glutelins accounting for 30% and prolamins, called kafirins (KAF) for the sorghum grain, represent 70–80% of the total protein.^1,3^ KAF are classified as α-KAF (25–23 kDa), β-KAF (20–16 kDa), γ-KAF (28–50 kDa), and δ-KAF (13 kDa) and are located in the endosperm, forming agglomerations and interacting with the glutelin matrix and distributed around the starch granules, which makes it difficult to extract KAF.^2–6^ The most applied extraction protocol to recover KAF is the use of ethanol and sodium metabisulfite as reducing agent,^7,8^ however, detailed information on suitable extraction methods of KAF using enzymatic pretreatments are limited.

In some studies, the starch in rice and corn flour has been hydrolyzed, in a process called liquefaction to increase the percentage of flour protein. In the liquefaction process, the starch granules that are interacting with the protein, can be hydrolyzed by the amyloglucosidase enzyme (EC 3.2.1.3). This enzyme hydrolyzes the α-1,4 glycosidic bonds from amylose and amylopectin, and also can rapidly hydrolyze α-1,6 glycosidic bonds when the next in the sequence is α-1,4 bonds, and then separate and remove the starch from the flour.^9,10^ This process generates a high-protein flour, not toxic and food grade (Mesa–Stonestreet 2010), and allows to increase the extraction yield of KAF using the conventional extraction method.^11^

Several studies have shown that the bioactivity of grain proteins possess angiotensin-converting enzyme (ACE) inhibitors, and anti-inflammatory, antioxidant, cholesterol-lowering, anticancer, and antidiabetic effects, which are based on their sequences and their structures.^12,13^ In general, the presence of hydrophobic amino acids (Pro, Ala, Val, Gly, Met, Ile, and Trp) indicated a higher biological activity like antioxidant capacity, amino acids (aa) present in the main storage protein of sorghum grain, KAF.^14,15^ Previous studies have shown that KAF and KAF fragments have potential antioxidant, ACE inhibitor, and anti-inflammatory activities.^14–17^

However, with the use of bioinformatics tools, such as The BIOPEP-UWM database, which contains biologically active peptide sequences, and PeptideRanker, can be applied for the identification of different bioactive KAF peptides with potential benefits of human health as well as the identification of unexplored peptides with potential biological activity, produced by enzymatic hydrolysis *in silico* using subtilisin, thermolysin, and chymotrypsin, which cleaves the peptide bonds of hydrophobic aa.^18^

Therefore, the objective of this study was to optimize KAF extraction process applying a pretreatment using amyloglucosidase, to identify which provides the highest KAF yield, and additionally use BIOPEP-UWM and PeptideRanker to predict potential bioactive peptides from the white sorghum grain (Perla 101).

## Materials and Methods

### Raw material

Grains of the white sorghum (*S. bicolor* L. Moench, variety Perla 101) were obtained from the National Research Institute for Forestry, Agriculture, and Livestock (INIFAP), Campus Culiacan, Mexico. Sorghum grains were manually peeled and subjected to a milling (IKA mill universal model M20, Staufen im Breisgau, Germany) and sieved through a 250 μm mesh. The flour obtained was stored at 4°C until further analysis.

### Pretreatment and kafirin extraction

Sorghum flour was treated with amyloglucosidase (EC 3.2.1.3, protease from *Aspergillus niger*, 260 U/mL; Sigma-Aldrich, St. Louis, MO) before the KAF extraction. The response surface methodology (RSM) is a collection of mathematical and statistical techniques and can be used as a potential tool to evaluate experimentation during screening and optimization of models. In this study, RSM was applied using a complete factorial design 3^2^, in which the hydrolysis time factor (*t*) levels were 30, 60, and 120 min, for the factor enzyme/substrate ratios (E/S) were 0.05%, 0.10%, and 0.15% v/w (enzyme/flour). The response variable was the yield of KAF (g/100 g dry flour), which was determined upon completion of amyloglucosidase treatment, after ethanol extraction explained below. The concentration was 20% w/v (raw flour/0.5 M sodium acetate buffer, pH 4.5) for all the treatments.

The reaction mixture was pregelatinized at 90°C for 10 min before adding the enzyme according to the method of Shih and Kim,^19^ and Paredes-López et al.^20^ with modifications, and design conditions 3^2^ were applied immediately: (1) 30 min, 0.05% E/S; (2) 30 min, 0.1% E/S; (3) 30 min, 0.15% E/S; (4) 60 min, 0.05% E/S; (5) 60 min, 0.1% E/S; (6) 60 min, 0.15% E/S; (7) 120 min, 0.05% E/S; (8) 120 min, 0.1% E/S; and (9) 120 min, 0.15% E/S. The enzyme was inactivated on ice for 5 min at the end of the hydrolysis time of each of the treatment. The mixture was centrifuged at 9000 *g* for 30 min at 4°C to separate and remove the hydrolyzed starch from the flour.

The precipitates were stored to perform the KAF extraction. The extraction of the KAF fraction was carried out following the methodology of Espinosa-Ramírez and Serna-Saldívar,^7^ with some modifications. The precipitates were mixed with 900 mL of 70% ethyl alcohol (containing 0.35% w/v of sodium metabisulfite) and stirred for 1 h at 70°C. The supernatants were diluted to a 40% alcohol concentration by adding distilled water; they were stored at −20°C for 24 h to promote the KAF precipitation. After this period, the KAF were centrifuged at 3500 *g* for 15 min at 4°C.

The precipitates were placed in an extraction hood for 24 h to allow evaporation of the solvent and were stored at −20°C for later use. Additionally, the yield of KAF extraction with the pretreatment was compared with other extraction methods using 100 g dry sorghum flour with 900 mL of solvent; the first only using ethanol (70% v/v), the second using ethanol (70% v/v) + sodium metbisulfite (0.35% w/v), and the last using *t*-butanol (100%) + sodium matabifulfite (0.5% w/v).

Protein concentration of all samples was determined as described by Bradford,^21^ being a widely used technique for being simple, fast, cheap, and sensitive. Bovine serum albumin (Sigma-Aldrich) was used as standard at a concentration range of 0–1 mg/mL. The model proposed for response variable (*Y*) was:

$$Y_{\%KAF}=\beta_{0}+\beta_{1}t+\beta_{2}ES+\beta_{1,2}tES+ɛ$$

where *β*_0_ is the intercept, *β*_1_ and *β*_2_ are linear coefficients, *β*_1,2_ is the interaction coefficient, and *ɛ* denotes the experimental error. The parameters of the model were estimated by multiple linear regression using the Statistica v. 10 software (StatSoft, Palo Alto, CA).

### Sodium dodecyl sulfate–polyacrylamide gel electrophoresis

KAF electrophoretic analysis was performed in miniplates (Mini Protean III^®^ Model; Bio-Rad, Hercules, CA) according to the method of Laemmli,^22^ to separate the fractions from the KAF and to be taken to the mass spectrometer to ensure their identification. A concentrator gel and separation gel (4% and 14% w/v polyacrylamide, respectively) were used in the presence of sodium dodecyl sulfate (SDS). Fifteen microliters of each sample (3 mg/mL) was applied and the electrophoresis was carried out at 110 V for 90 min. The molecular weight (M_W_) of the bands was determined using a protein standard (SDS-PAGE Standards, Broad Range, Catalog No. 161-0374; Bio-Rad).

### Protein identification

The bands identified in the gel were cut and washed with ultrapure water and acetonitrile grade mass spectrometry. The digestion process was carried out with the enzyme Trypsin (mass grade), to generate cuts right where the aa lysine and arginine, still within the acrylamide matrix; samples were retrieved and desalted for individual injection in the mass spectrometer. The chromatography system corresponds to a nanoACQUITY-UPLC system (Waters), has a Pre column Symmetry C18, 100 Å (5 μm × 20 mm), reverse-phase chromatographic column nanoACQUITY UPLC Peptide BEH C18, 130 Å (150 mm × 75 μm, particle size 1.7 μm) coupled with a nanoESI ionization for protein identification. The run of each sample was carried out under the following linear gradient in two phases of circumvention, one hydrophilic (Water, A) with 0.1% formic acid and another hydrophobic (Acetonitrile, B) with 0.1% formic acid in a gradient elution analysis programmed as follows: 0 min, 3% (B); 0–1 min, 3% (B); 5–37 min, 9–80% (B); 37–38 min, 80–5% (B), 38–40 min, 5% (B). The acquisition interval in Mass mode (MS) was 400–2000 (*m/z*); for the acquisition in Mass/Mass mode (MS/MS) it was of 50–2000 (*m/z*).

The program of acquisition was by Data-Dependent Acquisition. With incollision parameters defined as a fragmentation energy ramp, low mass collision energy (CE) 15–45 electronvolts (eV), and high mass CE 15–55 eV. The acquisition of Reference standard or Lock Mass was performed every 30 s for a time of 1 s. As a reference standard (Lock Mass), Human Fibrinopeptide B (with a mass of 1,571.6852 was used) *m/z* at charge 1, for charge 2 it is 785.8426, *m/z* at a concentration of 200 fmol/μL, at a flow of 0.5 μL/min. Protein identification was performed based on the NCBI (green plants, Viridiplantae) database.

### *In silico* analysis, peptide ranking, and bioactivity prediction

The sequences of sorghum storage proteins identified by nanoLC-MS/MS were subjected to *in silico* analysis using the BIOPEP-UWM database to identify potential bioactive peptides and also the hydrolysis of these sequences *in silico* to produce using thermolysin (EC 3.4.24.27), subtilisin (EC 3.4.21.62), and chymotrypsin (EC 3.4.21.1) enzymes. The PeptideRanker (http://distilldeep.ucd.ie/PeptideRanker, in January 2020) was used to rank the predicted sequences of peptides according to bioactivity, and the occurrence frequency (A) of bioactive fragments with a potential activity in the protein sequences was calculated by the equation: A = a/*N*, a was the number of amino acid residue (aa) forming fragments with given activity in protein sequence, and *N* is the number of total aa of the protein identified by MS.^18^

### Statistical analysis

Results were reported as mean ± standard deviations. Significant differences among treatments were determined by analysis of variance and Fisher's least significant difference tests (*p* < 0.05) using the Statistica v. 10 software (StatSoft, Inc.).

## Results and Discussion

### Optimization of kafirins extract

In this study, to promote the increase in KAF extraction performance, the starch granules that interact with the protein were hydrolyzed with the enzyme amyloglucosidase. Starch is the main carbohydrate and sorghum component, ∼79% of the weight of the dry grain, it is a biopolymer constituted by glucose molecules, which form amylose and amylopectin strands. Amylose is a linear polymer with α-1,4 glycosidic bonds, while amylopectin is a highly branched polymer that contains α-1,4 bonds and α-1,6 bonds.^23,24^

The hydrolysis of starch is generally in three stages: gelatinization where water and heat interrupt the hydrogen bonds that hold together the starch granules, which swell until a partial rupture, leading to the dispersal of amylose and amylopectin in the solution; liquefaction is catalyzed by enzymes like α-amylase and amyloglucosidase (also called glucoamylase) that produces oligosaccharides that can be degraded in the third stage of saccharification, obtaining glucose and maltose.^24^ In this project, the first two stages (gelatinization and liquefaction) were mainly carried out, which promotes the release of the proteins of interest (KAF), which are surrounded and interacting by hydrogen bonds with the starch matrix, now hydrolyzed.

After the enzymatic hydrolysis of the starch, the KAF extraction of the supernatants was continued (precipitated sorghum flour), using the methodology of Espinosa-Ramírez and Serna-Saldívar,^7^ with modifications. The pretreatment over the sorghum flour was followed of KAF extraction with ethanol (70% v/v) and sodium metabisulfite (0.35% w/v). The ethanol interacts with kafirins, weakening the hydrophobic interactions among them, allowing their extraction.^11^ In addition, in our case sodium metabisulfite was used as a reducing agent to allow the breakdown of the disulfide bonds of cysteine present in KAF and thus avoid the formation of disulfide bridges between the γ-KAF molecules on the surface of protein bodies, increasing the availability of protein for extraction.^11^

**FIG. 1.**
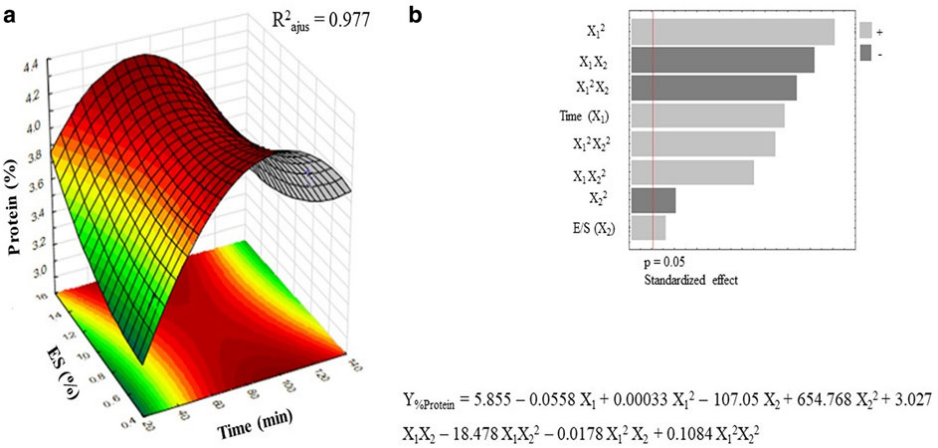
**(a)** Response surface plot indicating the effect of hydrolysis time (*t*) and enzyme/substrate ratio (E/S) of the pretreatment and extraction of the kafirin protein (%Protein). **(b)** Pareto chart and polynomial equation that fitted to the response surface model.

The response variable was the percentage of g of protein extract/100 g of dry flour using amyloglucosidase pretreatment (Y) measured for white sorghum flour under different combinations of enzyme/substrate ratio and time. Table 1 shows the experimental matrix design, with the results obtained. The response of KAF extract was analyzed using ANOVA. The response surfaces of kafirins extract Y1 and Y2 generated applying RMS with a second-order polynomial are shown in Figure 1a and b, respectively.

**Table 1.** Matrix of Experimental Design to Evaluate the Optimal Condition of the Pretreatment to Extract Kafirin Proteins

Run	Experimental factors		Response variable
	t (min)	E/S (%)	Protein (%)^*^
1	30	0.05	3.36 ± 0.100^e^
2	30	0.1	3.23 ± 0.013^d^
3	30	0.15	4.09 ± 0.06^a^
4	60	0.05	4.06 ± 0.033^a^
5	60	0.1	4.1 ± 0.004^a^
6	60	0.15	3.82 ± 0.020^c^
7	120	0.05	3.92 ± 0.03^b^
8	120	0.1	3.85 ± 0.025^b,c^
9	120	0.15	3.79 ± 0.020^b^

Values are shown as mean ± standard deviation of three replicates. Mean with different superscripts are significantly different (*p* < 0.05).

^*^ Grams protein/100 g of dry flour.

E/S, enzyme/substrate ratio.

The optimal pretreatment conditions using amyloglucosidase (*X*_1_ = 83 min, *X*_2_ = 0.086% w/v), with each conditions indicated by the model the predicted value was *Y* = 0.787 KAF g/extract g. To check the optimal extraction conditions, the experimental values were obtained, resulting in 0.743 KAF g/extract g. The models obtained has a satisfactory adjusted *R*^2^ of 0.977, demonstrating that it can be used to predict the extraction performances when applying the pretreatments.

The model for the amyloglucosidase presents a value *p* < 0.05 for the linear, quadratic, and the interactions between *X*_1_*X*_2_, the factor *X*_1_ have the most positive influence on the response variable, followed by interactions *X*_1_*X*_2_. In the response surface graph (Fig. 1), maximum point in factor *X*_1_ was observed around 60 min for the both models and *X*_2_ was between 0.05% and 0.10%, indicating that the amount of KAF is increasing as the variables increase, reaching the optimal value, but then the decrease in the value of KAF extracted by increasing *X*_1_ is observed. Finding that high liquefaction time (120 min) and enzyme/substrate concentrations (0.15% w/v) did not increase the KAF content in the extract (*p* < 0.05), amyloglucosidase is inhibited mainly by the generation of sugars, the presence of tannins, and polyphenols.^20,25^

White sorghum flour variety Perla 101 contains 8.38% ± 0.22% of total protein.^8^ The results indicate that the use of enzymatic processes with carbohydrases increased the extraction of the KAF, whose percentage varies between 69% and 73% of the total protein. In the Table 2, the different extraction methods used in the samples of sorghum grain variety Perla 101 are presented. The optimal pretreatment conditions were used with amyloglucosidase, obtaining 4.12% ± 0.02% of KAF. These results were compared with methods without using enzymatic pretreatment, where significantly lower values were observed (*p* < 0.05).

**Table 2.** Extraction Methods and Sorghum Grain *(Perla 101)* Protein Content

Protein total (%, db)	Extraction methods	Kafirin extract		
		Protein (%, g KAF/g of extract)	Protein yield (%, based on protein)	Global yield (%, based on protein total)
8.38 ± 0.22	*t*-butanol +0.5% (w/v) sodium metabisulfite^*^	45.4 ± 0.18^d^	29.83 ± 0.11^d^	2.5 ± 0.01^d^
	70% (v/v) aqueous ethanol	48.54 ± 1.43^c^	32.1 ± 0.95^c^	2.69 ± 0.07^c^
	70% (v/v) aqueous ethanol +0.35% (w/v) sodium metabisulfite	68.11 ± 0.63^b^	45.1 ± 0.41^b^	3.78 ± 0.03^b^
	Optimal pretreatment with amyloglucosidase (83 min, 0.086% E/S)^**^	74.2 ± 0.47^a^	49.16 ± 0.31^a^	4.12 ± 0.02^a^

Values are mean and standard deviations of three replicates, mean with different letters within a column differ significantly (*p* < 0.05).

^*^ Source: Castro-Jácome et al.^8^

^**^ Pretreatment followed by KAF extraction with 70% (v/v) ethanol and 0.35% (w/v) sodium metabisulfite.

Db, dry basis; KAF, kafirin.

The cases of the extractions with only ethanol and *t*-butanol, with values being ≤3.78% of protein. This indicates that when applying the pretreatment plus the extraction, an increase of the extraction yield of KAF was achieved of 64.8% for the variety Perla 101 using amyloglucosidase compared with what was previously reported by Castro-Jácome et al.^8^ In the present study, a maximum value of 4.12% ± 0.02% of KAF was obtained when using a pretreatment with amyloglucosidase, being similar to that provided by Espinosa-Ramírez and Serna-Saldívar,^7^ 4.24% ± 0.14% KAF, for whole regular white sorghum grains, finding that there is no significant difference (*p* < 0.05).

However, if there is a significant difference (*p* < 0.05) in the percentage of KAF extract obtained from decorticated white sorghum, this is 5.00% ± 0.11% KAF. This difference can be attributed to the fact that the purity of the protein and the yield depend on the genotype of the grain of sorghum and the texture of the endosperm, and not necessarily on the amount of total protein. Several authors conclude that KAF obtained from whole grains, as is our case, has lower extraction yields due to the presence of fat, polyphenols, and polysaccharides that are extracted simultaneously with KAF in the ethanolic process.^26,27^

The yield of extraction of KAF in the variety Perla 101, coincides with that reported by other authors in red sorghum grains (varieties SB-2413 and Maravilla) and white sorghum (varieties S-23, Mazatlán, Costeño, among others) with values between 1.44 and 4.65 g of protein/100 g of defatted flour, using *t*-butanol (40–90%) and 70% ethanol.^28^ Being the method of extraction with ethanol (after pretreatment with amyloglucosidase, 4.12 ± 0.02 g of protein/100 g of flour) applied in this study, it is the most environmentally friendly method, food grade, and therefore safer for its industrial application compared with that used with *t*-butanol. In addition, previous studies have used 2-mercaptoethanol (2-ME) at concentrations of 0.5–5% (v/v) as a reducing agent,^29–32^ allowing a yield of up to 5.42 g/100 g (using 70% *t*-butanol, three extractions and 5% 2-ME) to be achieved. However, based on the above, in the present study it was decided not to use 2-ME due to its high toxicity, using sodium metabisulfite as a reducing agent to allow the disulfide bonds of the cysteine present in the KAF to break down.^11^

### SDS–polyacrylamide gel electrophoresis of kafirins

**FIG. 2.**
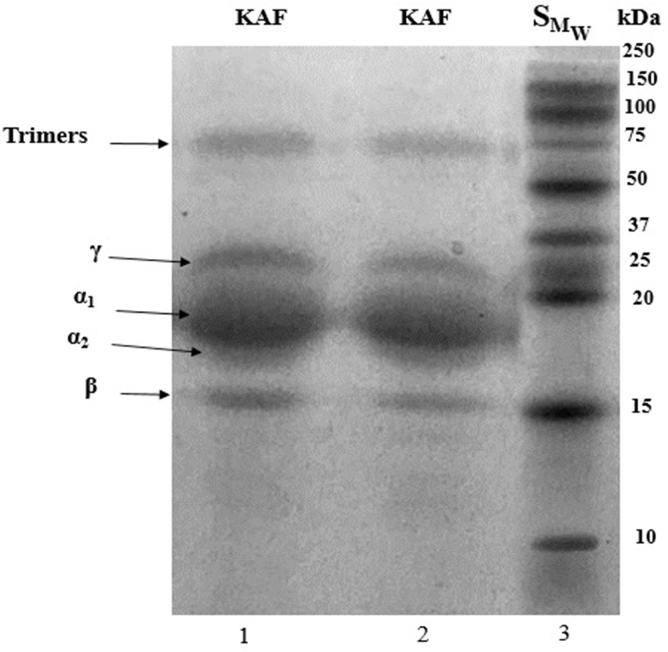
SDS-PAGE profile of kafirin from sorghum grain (variety Perla 101) with pretreatment. Lanes 1 and 2: kafirin extract (KAF), lane 3: protein standard (SM_W_). SDS-PAGE, sodium dodecyl sulfate–polyacrylamide gel electrophoresis.

KAF are classified based on their structure, solubility, and molecular weight and amino acid composition in four groups: α-KAF (23–28 kDa), β-KAF (18 kDa), γ-KAF (20 kDa), and δ-KAF (13 kDa).^14,26^ Figure 2 shows that the electrophoresis profile (SDS–polyacrylamide gel electrophoresis [SDS-PAGE]) was consistent with previous reports using reducing conditions,^7,8,14^ finding the presence of α_1_-, α_2_-, β-, γ-KAF, and a faint band around 100 kDa. Our results were within the bands reported for the Perla 101 grain without pretreatment with amyloglucosidase.

The most abundant fraction is α-KAF, representing 70% of the total prolamins and are located in the core of the protein bodies. According to Shull et al.^33^ α-KAF present two subunits: α_1_ and α_2_ with M_W_ between 24–28 and 22–23 kDa, respectively. The electrophoretic profile also showed a band of 17 kDa, which has been identified as β-KAF. According to Cremer et al.^34^ the band found around 35 kDa could be γ-KAF, because γ-prolamins are identified in maize and others plants. Another group of bands was observed in the region of 100 kDa, several authors indicate that these bands are generally identified as trimers or oligomers that could not be hydrolyzed with the reducing conditions.^3,7^

### Protein identification and predicted biological activity of sorghum grain peptides

Table 3 shows the aa sequences identified by nano-scale liquid chromatography coupled to tandem mass spectrometry from four bands obtained in the electrophoretic profile, β-kafirin = simple 4, α_2_-KAF = sample 2, α_1_-KAF = sample 3, and γ-KAF = sample 1. Sample 1 and 3 were associated with α-kafirin protein, accession number ACA28705.1 (*p* < 0.01), with sequence R.VNPVVAANPLAAAFLQQQQLLPFNQISILMNPAFSWQQPIVGSAIF, and sample 2 and 4 were associated with α-KAF protein precursor, accession number ACF19807.1 (*p* < 0.01 and *p* < 0.05, respectively), with a sequence R.LGAVSPATFWPQQQLLR.F, from *S. bicolor*. These results confirm that the KAF are obtained using the optimal pretreatment in conjunction with ethanol extraction.

**Table 3.** *Sorghum bicolor* Protein Identified Through Nano-Scale Liquid Chromatography Coupled to Tandem Mass Spectrometry

Sample	Protein name	Accession No.	Sequence	M_w_ (kDa)	Score	SPI %
**1**	α-KAF	ACA28705.1	R.VNPVVAANPLAAAFLQQQQLLPFNQISILMNPAFSWQQPIVGSAIF	29.0	72	16
**2**	α-KAF precursor	ACF19807.1	R.LGAVSPATFWPQQQLLR.F	25.8	85	7
**3**	α-KAF	ACA28705.1	R.VNPVVAANPLAAAFLQQQQLLPFNQISILMNPAFSWQQPIVGSAIF	29.0	100	16
**4**	α-KAF precursor	ACF19807.1	R.LGAVSPATFWPQQQLLR.F	25.8	88	7

The score of the ions is −10 × log (*p*), where *p* is the probability observed in the random event. The individual score of the ions must be greater than 50 (score >50), this indicates identity or extensive homology (*p* < 0.01). Gaps: spaces (.) for deletions and insertions. Databases NCBInr, Viridiplantae.

Once the proteins were identified (α-KAF and α-KAF precursor), the fragment sequences obtained (R.VNPVVAANPLAAAFLQQQQLLPFNQISILMNPAFSWQQPIVGSAIF and R.LGAVSPATFWPQQQLLR.F) were processed in the BIOPEP-UWM database to find peptides with biological activity. The computational studies conducted involved the comparison of MS-generated protein fragment sequences against peptides with reported bioactivity using a database search tool BIOPEP-UWM *in silico*.

Table 4 shows different biological peptide sequences that were found in the two protein sequences (α-KAF and α-KAF precursor), including: ACE-inhibitors, dipeptidyl peptidase IV (DPP-IV) and dipeptidyl peptidase III (DPP-III) inhibitors, antioxidative, hypotensive, stimulating, HMG-CoA reductase inhibitor, and activating ubiquitin-mediated proteolysis. The occurrence frequency (A) of bioactive fragments with a potential activity in the protein sequences was calculated (Table 4).^35^

**Table 4.** Bioactive Peptide Sequences Obtained from BIOPEP-UWM Database

Protein identified	Activity	Occurrence frequency (A)^a^	Sequences with biological activity	Description
α-Kafirin	ACE inhibitor	0.38	VAA, LAA, FNQ, LLP, LQQ, PL, AF, LA, AA, IF, VG, GS, MNP, LQ, AI, VNP, LPF, AFL	ACE inhibitor
	Glucose uptake-stimulating peptide	0.04	IV, LL	Stimulating
	Antioxidative peptide	0.02	LLPF	Antioxidative
	Ubiquitin-mediated-proteolysis activating peptide	0.02	LA	Activating ubiquitin-mediated proteolysis
	DPP-IV inhibitor	0.57	VA, LA, PA, LP, LL, VV, NP, QP, FL, SL, AA, PL, WQ, AF, FN, LM, MN, NQ, PF, PI, PV, QI, QL, QQ, SW, VG, VN	DPP-IV inhibitor
	HMG-CoA reductase inhibitor	0.02	IVG	HMG-CoA reductase inhibitor
	DPP-III inhibitor	0.06	LA, FL, PF	DPP-III inhibitor
23 kDa kafirin precursor	ACE inhibitor	0.35	VSP, GA, LG, PQ, TF, AV, LR	ACE inhibitor
	Glucose uptake-stimulating peptide	0.05	LL	Stimulating
	Antioxidative peptide	0.05	LLR	Antioxidative
	Renin inhibitor	0.1	LR, TF	Hypotensive
	DPP-IV inhibitor	0.6	PA, LL, WP, SP, GA, AT, AV, PQ, QL, QQ, TF, VS	DPP-IV inhibitor
	DPP-III inhibitor	0.1	LR, TF	DPP-III inhibitor

^a^ A = a/*N*, a was the number of amino acid residue-forming fragments with given activity in protein sequence, and *N* is the number of total aa of the protein identified by MS.

ACE, angiotensin-converting enzyme.

The α-KAF protein was high in DPP-IV inhibitors such as ACE inhibitors. Also α-KAF protein contained a small amount of DPP-III inhibitors (*A* = 0.06), stimulating (*A* = 0.04), antioxidative, HMG-CoA reductase inhibitor, and activating ubiquitin-mediated proteolysis with *A* = 0.02. Likewise, α-KAF precursor was high in DPP-IV inhibitors (*A* = 0.6) and ACE inhibitors (*A* = 0.35). Hypotensive, DPP-III inhibitors, antioxidative and stimulating were present in a smaller proportion.

In both sequences (α-KAF and α-KAF precursor) shows the higher occurrence frequency of potential peptides with DPP-IV inhibitors (*A* = 0.57 and 0.6), and ACE-inhibitors (*A* = 0.38 and 0.35). In addition, the BIOPEP-UWM tool was used to identify proteins by the hydrolysis performed *in silico*. Manoharan et al.^36^ used the BIOPEP-UWM database to predict *in silico* four stable tripeptides derived from mycelium of *Pleurotus pulmonarius* with ACE inhibitor potential using trypsin, chymotrypsin, and pepsin.

Wang et al.^37^ used the sequence of different proteins of animal origin (β-lactoglobulin, α-casein, β-casein, κ-casein, myoglobin) to predict possible peptides with DPP-IV inhibitory activity applying trypsin and pepsin. Han et al.^38^ carried out *in silico* hydrolysis of oilseed proteins such as sunflower and sesame, using subtilisin and pepsin, identifying peptides with DPP-IV inhibitory activity. In other studies, *in silico* the prediction of peptides with opioid, antioxidant, antimicrobial, and antithrombotic activity among others was achieved.^39,40^

In these studies, we have clearly demonstrated that *in silico* approach is reliable for predicting peptides with different biological activities of protein hydrolysates.

Table 5 shows the peptides formed by chymotrypsin, enzyme that cleaves peptide bonds selectively on the carboxyl-terminal side of the large hydrophobic aa such as tryptophan, tyrosine, phenylalanine, and methionine; subtilisin, it shows high specificity for aromatic and hydrophobic aa in the P1 substrate position, and thermolysin enzyme, cleaves peptide bonds at N-terminal of hydrophobic aa, including alanine, leucine, methionine, tyrosine, and valine.^41^ In addition, combinations of enzymes (chymotrypsin+thermolysim and chymotrypsin+subtilisin) were performed to increase the degree of theoretical hydrolysis (DH_t_%), obtaining higher DH_t_% (75% and 60%, respectively) with the combination of chymotrypsin+thermolysin, observing a higher production of peptides and aa for both sequences.

**Table 5.** Enzymatic Peptide Production Through *in silico* Approach Using BIOPEP-UWM Database

Protein sequence	Protein name	Enzyme action									
		Thermolysin		Subtilisin		Chymotrypsin		Chymotrypsin+Thermolysin		Chymotrypsin+Subtilisin	
		Results peptides and aa	DH_t_%	Results peptides and aa	DH_t_%	Results peptides and aa	DH_t_%	Results peptides and aa	DH_t_%	Results peptides and aa	DH_t_%
R.VNPVVAANPLAAAFLQQQQLLPFNQISILMNPAFSWQQPIVGSAIF	α-Kafirin	R. - VNP - V - V - A - ANP - L - A - A - A - F - LQQQQ - L - LP - FNQ - IS - I - LMNP - A - FSWQQP - I - VGS - A - I – F	50	R. - VNP - V - VAANPL - AAAF - L - QQQQL - L - PF - NQIS - IL - MNPAF - S - W - QQPI - VGS - AIF	35.41	R.VN - PVVAAN - PL - AAAF - L - QQQQL - L - PF - N - QISIL - M - N - PAF - SW - QQPIVGSAIF -	31.25	R. - VN - P - V - V - A - AN - P - L - A - A - A - F - L - QQQQ - L - L - P - F - N - Q - IS - I - L - M - N - P - A - F - SW - QQP - I - VGS - A - I – F	75	R. - VN - P - V - VAAN - PL - AAAF - L - QQQQL - L - PF - N - QIS - IL - M - N - PAF - S - W - QQPI - VGS - AIF	45.83
R.LGAVSPATFWPQQQLLR.F	α-Kafirin precursor	R. - LG - A - VSP - AT - FWPQQQ - L - LR. – F	40	R.L - GA - VS - PATF - W - PQQQL - L - R.F	35	R.L - GAVSPATF - W - PQQQL - L - R.F	25	R. - L - G - A - VSP - AT - F - W - PQQQ - L - L - R. – F	60	R.L - GA - VS - PATF - W - PQQQL - L - R.F	35

DH_t_%, theoretical degree of hydrolysis given by BIOPEP-UWM database.

In Table 6, PeptideRanker was performed, it can predict the probability of a peptide being bioactive according to their score between 0 and 1. Generally, any peptide over 0.5 threshold is labeled to be bioactive. In Table 6, the ranking of each peptide obtained *in silico* is shown, the peptides that presented the highest number (peptide ranking > 0.5, bold value) from the α-KAF protein using only thermolysin were LP (0.79, DPP-IV) and FNQ (0.61, ACE inhibitor), from α-KAF precursor was LG (0.71, ACE inhibitor). When only subtilisin was used, the potential peptide from the α-KAF precursor was PF (0.99, DPP-IV), and from α-KAF precursor was GA (0.52, ACE inhibitor). With only chymotrypsin, the potential peptide from the α-KAF protein was PL (0.81, ACE inhibitor), PF (0.99, DPP-IV inhibitor), and SW (0.93, DPP-IV), from α-KAF precursor none was reported (no data).

**Table 6.** Bioactivity of Predicted Peptide Sequences and Ranking from Peptides Production *in silico* Obtained by BIOPEP-UWM Database and PeptideRanker

Enzyme	Protein sequence	Know Peptides	Know activity	Peptide ranking
Thermolysin	R.VNPVVAANPLAAAFLQQQQLLPFNQISILMN PAFSWQQPIVGSAIF	LP	Dipeptidyl peptidase IV inhibitor	**0.79**
		FNQ	ACE inhibitor	**0.61**
		VNP	ACE inhibitor	0.17
	R.LGAVSPATFWPQQQLLR.F	VSP	ACE inhibitor	0.16
		LG	ACE inhibitor	**0.71**
		AT	Dipeptidyl peptidase IV inhibitor	0.07
Subtilisin	R.VNPVVAANPLAAAFLQQQQLLPFNQISILMN PAFSWQQPIVGSAIF	IL	Stimulating	0.39
		PF	Dipeptidyl peptidase IV inhibitor	**0.99**
		VNP	ACE inhibitor	0.17
	R.LGAVSPATFWPQQQLLR.F	GA	ACE inhibitor	**0.52**
		VS	Dipeptidyl peptidase IV inhibitor	0.04
Chymotrypsin	R.VNPVVAANPLAAAFLQQQQLLPFNQISILMN PAFSWQQPIVGSAIF	PL	ACE inhibitor	**0.81**
		PF	Dipeptidyl peptidase IV inhibitor	**0.99**
		SW	Dipeptidyl peptidase IV inhibitor	**0.93**
	R.LGAVSPATFWPQQQLLR.F	NO DATA	NO DATA	NO DATA
Chymotrypsin+Thermolysin	R.VNPVVAANPLAAAFLQQQQLLPFNQISILMN PAFSWQQPIVGSAIF	SW	Dipeptidyl peptidase IV inhibitor	**0.93**
		VN	Dipeptidyl peptidase IV inhibitor	0.04
	R.LGAVSPATFWPQQQLLR.F	VSP	Dipeptidyl peptidase IV inhibitor	0.16
		AT	Dipeptidyl peptidase IV inhibitor	0.07
Chymotrypsin+Subtilisin	R.VNPVVAANPLAAAFLQQQQLLPFNQISILMN PAFSWQQPIVGSAIF	PL	ACE inhibitor	**0.81**
		IL	Stimulating	0.39
		PF	Dipeptidyl peptidase IV inhibitor	**0.99**
		VN	Dipeptidyl peptidase IV inhibitor	0.04
	R.LGAVSPATFWPQQQLLR.F	GA	ACE inhibitor	**0.52**
		VS	Dipeptidyl peptidase IV inhibitor	0.04

Values in bold represent a peptide ranking > 0.5.

However, when performing the *in silico* combination of chymotrypsin and thermolysin, the known peptides from the α-KAF protein was SW (0.93, DPP-IV inhibitor), from the α-KAF precursor the two peptides VSP and AT have a peptide ranker <0.5. Finally, performing with chymotrypsin and subtilisin the known peptides from the α-KAF protein were PL (0.81, ACE inhibitor) and PF (0.99, DPP-IV inhibitor), and from α-KAF precursor was GA (0.52, ACE inhibitor). DPP-IV is a serine protease and glucagon-like peptide 1 (GLP-1) that degrades incretin hormones and a glucose-dependent insulinotropic peptide that affects β cells of the pancreas and leads to decreased insulin secretion, therefore DPP-IV is a therapeutic target for type II diabetes.

In 2006, DPP-IV inhibitor was used for the first time as a diabetic drug, and since then, different types have been approved to treat hyperglycemia. DPP-IV inhibitors prevent GLP-1 breakdown and restore normal insulin secretion and probably the peptide generation from KAF can be a viable alternative for the inhibitor production without side effects.^35–40,42,43^

On the other hand, ACE is an essential enzyme in the renin/angiotensin system that plays a vital role in controlling blood pressure and fluid balance. ACE raises blood pressure by catalyzing the reaction of the inactive decapeptide angiotensin-1 to the active vasoconstrictor angiotensin-2 causing high blood pressure and it is associated with increased heart failure, ischemic heart diseases, and coronary artery disease. Therefore, suppressing ACE is highly important.^13^ Barba de la Rosa et al.^44^ affirm that natural peptides have several vasodilator effects present in the sequences of α-KAF and α-KAF precursor (VAA, LAA, FNQ, LLP, LQQ, VSP), and these peptides can be a potential benefit in the prevention of cardiovascular diseases.

In addition, Kamath^45^ reports that α-KAF hydrolysates produced with chymotrypsin have ACE inhibitory effects. For all the above, using proteolytic enzymes, such as chymotrypsin, subtilisin, and thermolysin and their combination, could generate peptides derived of KAF with ACE, DPP-III, DPP-IV inhibitory activity and in less quantity with antioxidant, stimulating, hypotensive activity, being the stimulating and hypotensive activity the least explored using peptides derived from white sorghum grain. Likewise, it was possible to identify potential novel unexplored peptides sequence such ANP, LQQQQ, IS, LMNP, VGS, VAANP, AAAF, QQQQL, NQIS, MNPAF, QQPI, AIF, VN, PVVAAN, QISIL, PAF, QQPIVGSAIF, AN, QQQQ, QQP, VAAN, QIS, QQPI, LR, PQQQL, PQQQ, RF, RL, PATF, FWPQQQ, and GAVSPATF, taking as evidence the bioinformatic study using BIOPEP-UWM. However, more research is needed to generate and verify the beneficial effects of these peptides derived from KAF.

## Conclusion

Our results, based on amino acid sequences from KAF extracted with amyloglucosidase pretreatment and ethanol extraction, and hydrolyzed *in silico* using subtilisin, thermolysin, and chymotrypsin, indicate that KAF proteins may be good source for bioactive peptides, in particular for ACE inhibitor, DPP-IV inhibitor, DPP-III inhibitor, and antioxidant. These bioactive peptides could be useful ingredients for functional foods, and further studies are highly warranted to validate the predictions, in particular to confirm the potential biological activity from peptides that are currently not described, and establish their overall relevance for beneficial health *in vitro* and *in vivo*.

## Abbreviations Used

2-ME: 2-mercaptoethanol
ACE: angiotensin-converting enzyme
CE: collision energy
DPP: dipeptidyl peptidase
E/S: enzyme/substrate ratio
eV: electronvolts
KAF: kafirins
RSM: response surface methodology
SDS-PAGE: sodium dodecyl sulfate–polyacrylamide gel electrophoresis
